# Effects of functional physical activity on the maintenance of motor function in Alzheimer’s disease

**DOI:** 10.1590/S1980-57642009DN20300013

**Published:** 2008

**Authors:** Laís Fajersztajn, Renata Cereda Cordeiro, Solange Andreoni, Jacqueline Takayanagi Garcia

**Affiliations:** 1 Physiotherapist, Specialist in Gerontological Rehabilitation, Universidade Federal de São Paulo/Escola Paulista de Medicina (UNIFESP/EPM).; 2 MSc, physiotherapist, Coordinator of the Gerontological Rehabilitation Department of the Lar Escola São Francisco, UNIFESP/EPM, São Paulo, Brasil.; 3 PhD, Adjunct Professor of Biostatistics, Department of Preventive Medicine, UNIFESP/EPM, São Paulo, Brasil.; 4 MSc, Physician, Gerontological Rehabilitation Department of the Lar Escola São Francisco, Department of Preventive Medicine, UNIFESP/EPM, São Paulo, Brasil.

**Keywords:** motor function, Alzheimer’s disease, balance, physical activity, physiotherapy

## Abstract

It is widely known that older adults, even frail individuals, can improve their physical function using appropriately targeted exercise. Nevertheless, older adults with Alzheimer’s disease (AD) have been excluded from the majority of studies on exercise. The functional-task physical activity program is based on activities of daily living, and may be suited for elderly people with AD because it focuses on the maintenance and stimulation of preserved abilities. In addition, session costs are substantially reduced by adopting a group approach. Furthermore, the group approach may improve the social interaction of the demented patient.

**Objectives:**

To determine whether a functional-task physical activity program in groups can maintain motor function in elderly with AD.

**Methods:**

10 elderly diagnosed with mild or moderate AD were assigned into one of two groups: subjects with and without intervention. The intervention consisted of a 12-week function-task physical activity program in groups. Measurements: activities of daily living (Katz and Lawton & Brody questionnaires), mobility (Timed Up and Go Test, Timed Up and Go manual Test and Timed Up and Go Cognitive Test), cognition (Mini-Mental State Examination), behavioral disturbances (Neuropsychiatric Inventory I-brief) and functional balance (Berg Balance Scale).

**Results:**

A statistically significant difference between the two groups was found regarding the functional balance mean change measured by Berg scale score (p=0.046). A significant improvement of 1.60 points (95%CI[0.22;2.98]) was observed in the intervention group on this scale, while the non-intervention group showed –0.40 points (95%CI[–1.78;0.98], no change).

**Conclusions:**

It is possible to treat mild and moderate Alzheimer’s patients using a group approach. The functional task physical activity program was efficient in functional balance improvement and also appeared to prevent mobility decline.

## Abstract (pt)

Apesar dos conhecidos benefícios dos exercícios físicos resistidos em idosos frágeis, os idosos com doença de Alzheimer (DA) vêm sendo excluídos destes estudos. A atividade física funcional, por direcionar os exercícios para atividades comuns no dia a dia, seria adequada a este público, por enfocar a manutenção e a estimulação das capacidades remanescentes. A abordagem em grupo seria adequada tanto para reduzir os custos, quanto para atuar no isolamento social.

**Objetivos:**

Verificar os efeitos da atividade física funcional em grupo sobre a manutenção da função motora do paciente ido so com DA como forma de reabilitação preventiva.

**Métodos:**

10 idosos com DA leve a moderada alocados em dois grupos: com intervenção (atividades motoras com enfoque funcional durante 12 semanas) e sem intervenção. Foram avaliadas: atividades básicas e instrumentais de vida diária (questionário de Katz e de Lawton & Brody), mobilidade (*Timed Up and Go Test, Timed Up and Go Test manual e Timed Up and Go Test* cognitivo), cognição (Mini-exame do estado mental) e comportamento (Inventário Neuropsiquiátrico: NPI-Brief). O equilíbrio foi avaliado pela escala Berg de equilíbrio (BBS).

**Resultados:**

Houve diferença estatisticamente significativa entre os dois grupos quanto à mudança média no equilíbrio medido pelo número de pontos na escala de Berg (p=0,046). Um aumento significativo de 1,60 pontos (IC95%[0,22;2,98]) foi observado no grupo com intervenção nessa escala, enquanto que no grupo sem intervenção foi de –0,40 (IC95%[–1,78;0,98], nenhuma mudança).

**Conclusões:**

É possível tratar DA em grupo com estratégias motoras. Essas estratégias se mostraram eficazes na melhora do equilíbrio, sugerindo que a atividade motora pode prevenir o declínio da mobilidade em portadores de DA leve a moderada.

Due to the cognitive, behavioral and social alterations caused by Alzheimer’s, motor dysfunction in this disease has received the least attention. It is widely described in the literature that motor dysfunction in Alzheimer’s disease (AD) is a late symptom and may appear in the form of extrapyramidal signs and gait disturbances, culminating in immobility.^1,2^ However, more recent studies have reported motor dysfunction even in early stages and mild forms of AD.^3-6^

Motor alteration can manifest in many forms and different stages of AD. Since gait and balance are complex activities, requiring integration of motor, sensorial and cerebellar processes,^3^ gait velocity reduction and balance deficit generally emerge early in the first stages of dementia prior to apparent cognitive deficiency.^6^ In contrast, simpler motor activity such as grip strength is reduced in the late stages of the disease when cognitive disability has become evident.^3^

Motor alteration in AD can affect posture and posture control, with a marked motor velocity reduction.^5-7^ This might be explained by an impairment of various sensory inputs^8^ coupled with delays in the activation of responses to postural perturbation.^6^ In a study carried out by Peterson and co-workers (2005)^4^ on 140 individuals, those with AD had a much slower performance on basic mobility tests (Timed Up and Go Test and Manual Timed Up and Go Test) compared to healthy individuals and those with mild cognitive disability.

Cautious gait is the most common pattern in these patients, associated to a real or perceived instability.^5,6,9^ Balance deficit can be the root cause, and may contribute to reducing complex psychomotor actions as well as general movements and activities.^6^ The cerebral mechanisms that compensate for the physiological alterations of posture control components in healthy elderly individuals may fail in those with a degenerative brain disease.^6^ Regardless of the etiology, balance loss has significant psychological and emotional repercussions that can generate anxiety, reduce physical activity and lead to loss of social contact, common in AD.^6^

In a study on 45 individuals with AD, postural sway and gait abnormalities, including decreased walking speed and stride length, were associated with reduced cerebral blood flow in the basal ganglia and frontal lobes.^10^

Elderly training in focused physical activity can improve cardiovascular function, flexibility balance and muscle.^11,12^ Physical activity prevents and reduces the risk of developing secondary conditions stemming from functional decline and disuse^13^ and may also improve performance on depression and inactivity scales for individuals with AD dementia.^11^ In a six-year follow-up study conducted by Wang and co-workers (2006)^3^ on 1422 elderly individuals, regular physical activity was associated to a lower risk of developing dementia, suggesting that physical exercise offers cognitive benefits due to the connection between cognitive and motor functions.

Physical activity with functional focus has been associated to a reduction in dependence and disability among elderly individuals.^13^ Functional exercise can work muscle groups at the same time and in an integrated fashion, especially for daily functional activity movements. In a pilot study, a functional-task exercise program proved both feasible and well tolerated by community living older women.^14^

Despite the known benefits of resistance exercise in frail elderly individuals, those with dementia have been excluded from these studies.^12,13^ Considering these results and the consistently strong association between physical exercise and health in elderly individuals without dementia,^15^ we should not overlook the potential benefits of exercise for those with dementia.^11^

As the cognitive and behavioral alterations are exuberant, disease effects on motor function have not been extensively studied. Thus, motor function tends to be overlooked when assessing AD patients. Compared to same age controls, AD patients have worse physical performance,^16^ greater risk of falls and fractures^5-7^ besides faster mobility decline.^11^

Since the pharmacological therapy results are limited^1,2,17^ other approaches such as multidisciplinary management focused on maintenance and stimulation of the remaining abilities in individuals with AD have been gaining increasing interest for fulfilling the needs of these patients and their caregivers.^18^ It is believed that physical health can be improved, with a reduction in falls and the degree of fragility through intervention focused on motor function.^6,11,17^

AD patient balance and coordination may be maintained or even improved through focused intervention. Improvements in balance and posture control, together with boosted self-confidence during movement, lead to an improvement and optimization of function.^6^

Most AD individuals develop mobility problems and dependence for activities of daily living (ADL).^4,7^ Even the diagnosis of dementia leads to a reduction in the caregiver’s expectations regarding the patient and contributes toward deconditioning and premature mobility limitations due to an underestimation of the residual abilities of the patient.^17^ Thus, improving patient´s physical condition may extend their independence with regard to mobility, thereby im proving quality of life for themselves and caregivers, despite the progression of the disease.^4,11^

As described above, there are many benefits of enrolling AD patients in an exercise program. The group approach helps reduce costs and avoid social isolation. Thus, the aim of the present study was to determine the effects of functional physical activity in maintaining motor function in elderly patients with AD as a form of preventative rehabilitation.

## Methods

### Participants

Elderly individuals residing in the community and referred to the Gerontological Rehabilitation Department of the Lar Escola São Francisco – Universidade Federal de São Paulo / Escola Paulista de Medicina (UNIFESP/EPM) with AD diagnosis and willing to attend a rehabilitation program, participated in the study during the second semester of 2006. The Department of Geriatry reevaluated the patients and diagnosed AD based on the Diagnostic and Statistical Manual of Mental Disorders, IV edition (DSM-IV) of the American Psychiatric Association criteria^19^. Inclusion criteria were a score on the Mini-Mental State Exam (MMSE)^20^ between 10 and 26 and a Clinical Dementia Rating (CDR)^21,22^ of 1 or 2. All tests were administered twice by a trained, non-blinded evaluator. The exclusion criteria were clinical and motor conditions that contraindicated physical activity, lack of an informant for data collection and severe conditions that limited participation in the group, such as visual impairment, hearing impairment or behavioral disorder.

All family caregivers signed terms of informed consent. The Research Ethics Committee of the University approved the study, under protocol number CEP 1874/06, in compliance with Resolution 196/96 of the National Health Methodology Council.

### Experiment design

The individuals were consecutively allocated to two groups: intervention (first five patients to seek treatment in the department) and non-intervention (next five patients). Intervention took place over a 12-week period, with a single 1-hour session per week. Communication stimulation was carried out by specialized personnel one hour prior to each session. The non-intervention group awaited the call for the formation of the next group. All patients were evaluated at baseline and reevaluated at the end of the 12-week period. In order to be included in the study, each patient in the intervention group was required to attend at least 80% of the sessions.

### Evaluation

Evaluation included information from caregiver reports and observations on performance considering functional, motor, cognitive and behavioral aspects as well as socio-demographic data.

Activities of daily living (ADL) were assessed using the Katz index^23^ (score range from 0 to 6, higher scores denoting greater independence) while instrumental activities of daily living (IADL) were assessed using the questionnaire developed by Lawton and Brody (1969)^24^ (score range from 0 to 27, higher scores denoting greater independence). These instruments have not been validated in Brazil, but have been widely used in clinical practice in the country, including for patients with AD.^2,25^

Functional balance was assessed using the Berg Balance Scale (BBS).^26,27^ This scale consists of assessing an individual’s performance on 14 common tasks of daily living that challenge functional balance in an increasing degree of difficulty, ranging from remaining seated with no back rest, to picking up an object from the floor, reaching forward and standing on one foot. The score ranges from 0 to 56 points, higher scores denoting better performance. Mobility was assessed using the Timed Up and Go Test (TUG),^28^ which consists of determining the time in seconds for an individual to stand up from an armchair, walk 3 meters, turn around, go back and sit in the chair again. The individuals were also assessed using modified forms of the TUG. The Manual Timed Up and Go Test (manual TUG) is a modified version of the TUG in which a manual motor task (carrying a glass of water) is associated to the task in the original test.^29,30^ The Cognitive Timed Up and Go Test (cognitive TUG) is a modified version of the TUG in which a cognitive task (saying out loud the result of subtractions of 3 beginning with the number 20) associated to the task in the original test.^30^ For cognitive TUG performance, individuals were separated into two categories: yes (capable of performing the test) and no (incapable of performing the test).

Cognitive ability was assessed using the Mini-Mental State Examination (MMSE),^20^ whereas behavior was assessed using the Brief Clinical Form of the Neuropsychiatric Inventory (NPI-Q),^31,32^ which assesses 12 behavioral alterations (NPI FxS) as well the burden of the caregiver regarding these alterations (NPI Distress). This instrument has not yet been validated in Brazil.

### Intervention

The physiotherapy motor intervention program was designed as a group activity and consisted of physical activities with stimulation of balance and mobility through functional exercises. The same program was repeated in all sessions with progression of the exercises by adding cognitive tasks to the activity (e.g.: doing exercise at the same time as counting the repetitions or while holding an object) as well as by increasing the number of repetitions and load.

Sessions began with muscle stretching and joint movement. Muscle strengthening was carried out through functional activities (e.g. postural transference of objects from one place to another). Balance and physical conditioning were trained through walking, ball activities and circuit training with functional challenges (e.g. stairs and picking up an object from the floor). The sessions ended with stretching, massages and breathing exercises aimed at tactile stimulation, body awareness and relaxation.

### Statistical analysis

Statistical analysis was carried o ut using the SPSS program (version 13). Comparisons of the categorized variables between the two treatment groups at baseline (socio-demographic, functional, motor and cognitive data) were performed using Fisher’s exact test, whereas comparisons of the continuous variables were performed using Student’s t-test for independent samples.

Comparison of mean absolute changes in values on the MMSE,^20^ IADL,^24^ ADL,^23^ BBS,^26,27^ TUG,^28^ manual TUG^29,30^ and NPI^31,32^ scales between the two groups were performed through analysis of variance models for repeated measurements. Group type (intervention and non-intervention) was the *between* individuals factor, and time (baseline and endpoint) and group* time interaction were the *within* individual factors. Mean changes within each group were calculated using point estimates and 95% confidence intervals (95%CI). The level of significance was set at 5% for all statistical analyses.

## Results

All patients completed the experiment intervention. Adherence to the sessions was very satisfactory, with an average attendance of 88%. Weekly transportation of the patients to the service required a greater effort on the part of caregivers in some cases, but the evaluator noted good participation by patients in the activities and their apparent satisfaction at being able to perform them. No participants refused to perform any of the activities. In reevaluation interviews, caregivers reported that participating in the sessions had enabled them to improve patient management and learn what activities the patients were able to perform as well as their limitations. The caregivers reported satisfaction in seeing the patients engaged in activities and also reported that the patients w ere very satisfied in being able to perform activities by themselves in a specific social setting.

Tables 1 and 2 show the socio-demographic characteristics of the sample. These tables also present the values of the variables at baseline per group, which proved similar.

**Table 1:** Summary statistics of categorical variables for intervention and non-intervention groups at baseline.

Characteristic		Group								
		Non-Intervention (n=5)			Intervention (n=5)			Total n=10)		
		n	%		n	%		n	%	p*
Gender	Male Female	0 5	0% 100%		2 3	40% 60%		2 8	20% 80%	0.444
Falls	Yes No	3 2	60% 40%		4 1	80% 20%		7 3	70% 30%	1
Marital status	Married Not married	2 3	40% 60%		1 4	20% 80%		3 7	30% 70%	1
Caregiver	Formal Informal Both	1 4 0	20% 80% 0%		3 0 2	60% 0% 40%		4 4 2	40% 40% 20%	0.079
Household arrangement	Head or spouse, simple family Head or spouse, compound family Alone Neither head nor spouse	1 1 1 2	20% 20% 20% 40%		0 1 3 1	0% 20% 60% 20%		1 2 4 3	10% 20% 40% 30%	0.714
Schooling	Incomplete elementary Complete elementary Post-elementary	2 1 2	40% 20%4 0%		0 1 4	0% 20% 80%		2 2 6	20% 20% 60%	0.683
Ethnic background	Caucasian Non-caucasian	4 1	80% 20%		4 1	80% 20%		8 2	80% 20%	1
Nationality	Brazilian Non-Brazilian	4 1	80% 20%		3 2	60% 40%		7 3	70% 30%	1
CDR	Mild Moderate	4 1	80% 20%		2 3	40% 60%		6 4	60% 40%	0.524
Gait assistance device	Yes No	1 4	20% 80%		0 5	0% 100%		1 9	10% 90%	1
AD medication	Yes No	4 1	80% 20%		4 1	80% 20%		8 2	80% 20%	1
Fall risk†(BBS ≤45)	Yes No	2 3	40% 60%		4 1	80% 20%		6 4	60% 40%	1
Cognitive TUG	Yes No	1 4	20% 80%		3 2	60% 40%		4 6	40% 60%	0.524

* Fisher's exact test p;

† Fall risk ≥45.

**Table 2:** Summary statistics of continuous variables for intervention and non-intervention groups at baseline.

Variable	Statistic	Group		Total (n=10)	Student's t-test	p
		Non-Intervention (n=5)	Intervention (n=5)			
Age (years)	Mean SD^§^ Min-Max	76.40 7.50 68-87	78.40 6.43 70-87	77.40 6.67 68-87	-0.45	0.663
Weight (kg)	Mean SDMin-Max	52.26 10.31 43.3-70	69.42 10.25 57.5-82	60.84 13.26 43.3-82	-2.64	0.030
Height (cm)	Mean SD Min-Max	152.80 9.70 144-167	158.20 5.79 152.5-165	155.50 8.05 144-167	-1.07	0.316
MMSE	Mean SD Min-Max	19.80 2.39 17-23	20.60 5.94 11-26	20.2 4.29 11-26	-1.88	0.759
BBS	Mean SD Min-Max	47.40 4.88 42-53	47.80 2.95 43-50	47.60 3.81 42-53	-1.57	0.879
TUG (seconds)	Mean SD Min-Max	15.54 5.27 9.4-22.39	13.53 3.72 8.31-17.03	14.53 4.43 8.31-22.39	0.70	0.506
Manual TUG (seconds)	Mean SD Min-Max	17.13 5.21 9.5-22.4	14.68 3.94 8.84-19	15.91 4.54 8.84-22.4	0.84	0.425
NPIDistress	Mean SD Min-Max	19.40 9.56 9-29	17.20 9.55 6-30	18.30 9.0 86-30	0.36	0.725
NPI FxS	Mean SD Min-Max	57.00 34.79 20-96	49.00 25.39 20-87	53.02 9.02 20-96	0.42	0.689
ADL (Katz)	Mean SD Min-Max	5.20 1.10 4-6	4.40 1.34 3-6	4.80 1.23 3-6	1.03	0.332
IADL (Lawton)	Mean SD Min-Max	16.80 3.03 15-22	13.60 6.43 10-25	15.20 5.03 10-25	1.01	0.343

SD^§^, standard deviation.

**Graph 1:**
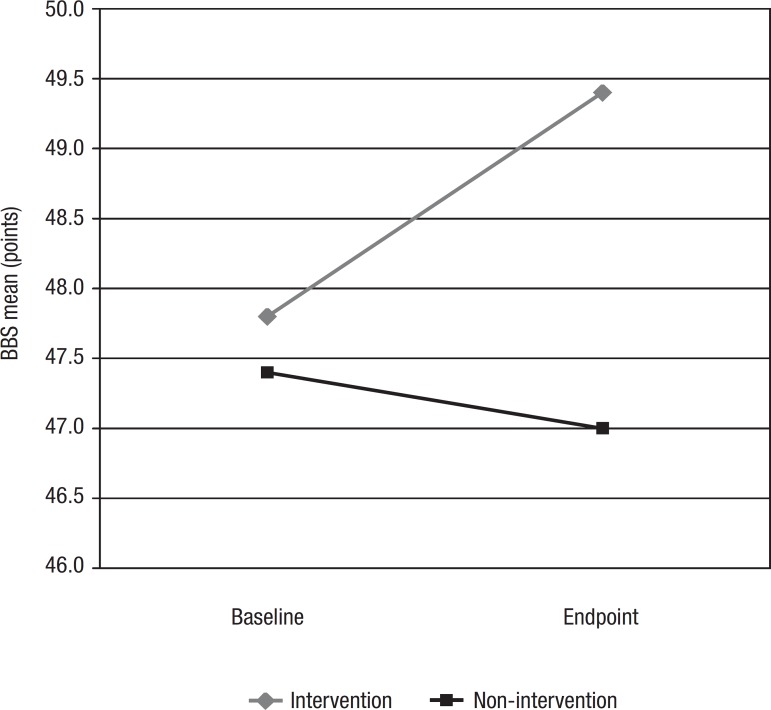
Berg Balance Scale (BBS) mean scores at baseline and endpoint.

Table 3 shows a summary of the results in the intervention and non-intervention groups. No statistically significant differences were found between groups regarding mean changes on the MMSE, NPI-Q, ADL and IADL scales. There were also no statistically significant differences between the two groups for mean changes in functional range on the TUG and manual TUG. However, a statistically significant difference between the two groups (p=0.046) was found for mean changes in functional balance measured by the number of points on the BBS scale. This difference is explained by the fact that a significant improvement of 1.60 points (95%CI[0.22;2.98]) was observed in the intervention group, while no change occurred in the non-intervention group (–0.40 points, (95%CI[–1.78;0.98]). The BBS mean profile by evaluation time and intervention group is depicted in Graph 1.

**Table 3:** Summary of results for intervention and non-intervention groups at baseline and endpoint.

Variable	Time	Group									
		Non-Intervention				Intervention				Change comparison	
		Mean	SE	95%CI		Mean	SE	95%CI		F	p
MMSE	Baseline Final Change	19.80 18.20 -1.60	2.03 2.65 1.03	-3.96 0.76		20.60 21.40 0.80	2.03 2.65 1.03	-1.56 3.16		2.74	0.136
BBS	Baseline Final Change	47.40 47.00 -0.40	1.80 2.06 0.60	-1.78 0.98		47.80 49.40 1.60	1.80 2.06 0.60	0.22 2.98		5.56	0.046
TUG(seconds)	Baseline Final Change	15.54 15.65 0.12	2.04 2.19 0.88	-1.92 2.15		13.53 13.27 -0.26	2.04 2.19 0.88	-2.29 1.77		0.09	0.770
Manual TUG(seconds)	Baseline Final Change	17.13 19.44 2.30	2.07 2.79 1.66	-1.53 6.13		14.68 14.45 -0.23	2.07 2.79 1.66	-4.06 3.60		1.16	0.312
NPI Distress	Baseline Final Change	19.40 19.00 -0.40	4.27 3.54 2.78	-6.80 6.00		17.20 14.60 -2.60	4.27 3.54 2.78	-9.00 3.80		0.31	0.591
NPI FxS	Baseline Final Change	57.00 55.00 -2.00	13.62 10.82 8.88	-22.47 8.47		49.00 42.20 -6.80	13.62 10.82 8.88	-27.27 13.67		0.15	0.712
ADL Katz	Baseline FinalChange	5.20 5.20 0.00	0.55 0.55 0.00	- -		4.40 4.40 0.00	0.55 0.55 0.00	- -		-	-
IADLLawton	BaselineFinalChange	16.80 15.60 -1.20	2.25 2.42 0.52	-2.40 -0.01		13.60 13.60 0.00	2.25 2.42 0.52	-1.20 1.19		2.67	0.141

SE, standard error.

Although not statistically significant (p=0.1363; Table 3), a tendency was observed for MMSE scores to be maintained in the intervention group (95%CI [–1.56;3.16]) yet decrease in the non-intervention group (95%CI [–3.97;0.76]) (Table 3).

## Discussion

There is no evidence that patients with dementia can be rehabilitated and restore lost skills.^34^ This progressive illness has a slow, continuous course, with survival rates ranging from 15 to 20 years.^1,13^

The annual cost of an individual with AD in the United States reached $36.100,00 in 1996. By the year 2050, 14 million Americans are expected to have AD.^35^ In Brazil, an epidemiological study carried out in an urban area of the city of Catanduva, São Paulo by Herrera and co-workers (2002)^36^ revealed a 7.5% prevalence of dementia in the elderly population. The cost of AD is not merely financial, but human and emotional. Pharmacological options have shown results, albeit limited.^2,11,16^ In order to fulfill the needs of these patients and their families, a multidisciplinary approach addressing the remaining abilities appears promising.^2,18^ In the quest for better options, the present study was able to obtain an improvement in functional balance of patients with AD following motor intervention focused on functionality.

Corroborating our results, Franssen et al. (1999)^6^ stated that balance and coordination in patients with Alzheimer’s can be maintained and even improved through focused intervention.

A diagnosis of dementia leads to deconditioning and premature mobility limitations due to underestimation of residual abilities and reduced expectations regarding the patient on the part of caregivers.^17^ Thus, the improvement in functional balance achieved in the present study most likely did not stem from the restoration of a lost ability as a direct consequence of the pathology, but rather through stimulation by the caregivers of the limited remaining skills of the patients.

The lack of consensus on the tools for assessing motor function in patients with AD hinders comparisons with the few studies published on physical activity among individuals with dementia.^13^ After an analysis of the different assessment tools for motor function in individuals with dementia, Thomas et al. (2002)^17^ suggested the use of these instruments without any significant changes in their protocols. However, Pettersson et al. (2005)^4^ observed motor alterations in early AD and stated that current scales may not be sensitive enough to encompass the total range of the disease. Thus, we believe that there may have been other benefits from the intervention that went undetected by the scales employed.

A meta-analysis selected 30 studies^13^ involving 2020 individuals with cognitive deficiency and dementia, and found that physical exercise improved health, physical functioning, cognitive functioning and positive behavior in this patient group. Tappen et al. (2000)^12^ carried out a study involving 65 individuals in which a program of walking and talking for 30 minutes three times a week for 16 weeks proved efficient in preventing a reduction in functional mobility in institutionalized individuals with AD. However, Cott et al. (2002)^37^ performed a similar study in 103 individuals and did not achieve positive results. Teri et al. (2003)^11^ carried out a 24-month follow-up study with 153 individuals and concluded that the teaching of behavior management techniques to caregivers, associated to physical exercise training, improved physical health and behavior scores on the sub-items of two physical health-functional assessment scales: the 36-Item Short-Form Health Survey (SF-36) and the Sickness Impact Profile (SIP). The behavior of the patients with AD was assessed by a depression scale.

Satisfactory adherence to the exercise program was directly proportional to the motivation and presence of the caregiver, who reported that the greatest difficulty in bringing the patient was overcoming inertia and apathy regarding leaving the house. Although not assessed, particularities of individuals such as a higher level of schooling may have influenced the high degree of adherence to the intervention.

Tappen and co-workers (2000)^12^ discussed the importance of social interaction for patients with AD, pointing out that, even in advanced stages of the disease, patients desire interaction with others, even when initially resistant to the idea. Researchers have also stressed that a trained physiotherapy team can improve the level of physical activity in different populations through encouragement and realistic goals. This discussion advocates group interventions and stresses the importance of a specialized physiotherapist to conduct the intervention. It is essential for these healthcare professionals to develop and employ strategies directed at patients with AD, knowing how to motivate them and deal with conflictive situations between them.

Considering the high degree of adherence to the program, the present study suggests that it is possible to intervene in AD by grouping patients together, as found in a few earlier studies on cognitive rehabilitation.^2,25^

Despite the methodological limitations of the present study (small sample size and short intervention period), we observed benefits in functional balance in the intervention group. Our study indicates that it is possible to treat AD in a group situation using motor skill strategies. These strategies were efficient in improving balance, which suggests that motor activity may prevent the decline in mobility among patients with mild to moderate Alzheimer’s disease. Further controlled studies are needed on elderly individuals with mild to moderate AD, which focus on supervised group physical activities and investigate a larger sample over a longer period of time in order to confirm the improvement in balance as well as other possible gains. A consensus is also needed on assessment tools for motor skills in patients with dementia to aid comparison between studies.
